# Minimizing Medication Errors from Electronic Prescription Transmission—Digitizing Compounded Drug Preparations

**DOI:** 10.3390/pharmacy7040149

**Published:** 2019-11-07

**Authors:** Richard H. Parrish, Lucy Gilak, Donna Bohannon, Steven P. Emrick, Brian Serumaga, Roy Guharoy

**Affiliations:** 1 St. Christopher’s Hospital for Children, Philadelphia, PA 19134, USA; 2 School of Pharmacy, Virginia Commonwealth University, Richmond, VA 23284, USA; 3 College of Arts and Sciences, University of Pennsylvania, Philadelphia, PA 19104, USA; lucy.gilak@usp.org; 4 United States Pharmacopeial Convention, Rockville, MD 20852 USA; DZB@usp.org (D.B.); SPE@USP.org (S.P.E.); BNS@USP.org (B.S.); 5 Baptist Health System, Montgomery, AL 36116, USA; RGuharoy@baptistfirst.org; 6 School of Medicine, University of Massachusetts, Worcester, MA 01655, USA

**Keywords:** compounded drug preparation, electronic prescribing, health information technology, interoperability, medication error, systemized nomenclature of medicine

## Abstract

Lack of standardization related to compounded drug preparations, especially in the transition of care situations, threatens patient safety by facilitating medication error. This paper outlines progress to-date from the United States Pharmacopeia (USP) Expert Panel on the Exchange of Compounded Drug Preparation Information in Health IT Systems. The work plan developed for the group is focused on proposing a set of encoding rules that would govern how compounded nonsterile drug preparations (CNSPs) are digitized and exchanged, including patient electronic health records (EHR), pharmacy systems, e-prescribing (eRx), and other Health IT (HIT) systems to ensure a seamless compounding process tailored to the needs of an individual patient. Included in this work are identifying authorized compounding monographs, surveying provider and end-user groups for information about data specificity during e-prescribing, and generating guidelines for the development of a compatible data model for clinical formulation identifiers (CF-IDs). This paper will also discuss how evolving nomenclature standards for CNSPs within HIT systems are part of a quality assurance system for comprehensive medication management (CMM) in children, thereby minimizing medication errors across the continuum of care. Finally, a network approach for the design of medication management systems for children and their families/caregivers is proposed.

## 1. Introduction

Drug compounding occurs when products are combined, admixed, diluted, pooled, or otherwise altered in a way different from that provided on a manufacturer package insert to prepare a medication [1]. Unlike manufactured products approved by the United States Food and Drug Administration (FDA) or those found on the British National Formulary for Children [2], compounded nonsterile preparations (CNSPs) are not officially designated or represented in any electronic vocabulary standards that can be stored and transmitted into patient records repositories through e-prescription (eRx) processing systems. This lack of standardization leads to the incomplete transmission of pertinent information and poses a significant safety risk to patients. Especially, vulnerable populations, such as children with special healthcare needs (CSHCN) and those with medical complexity (CMC) are at great risk. In fact, one recent estimate of the magnitude of medication errors in children in the outpatient setting related to eRx functionality subsumed in electronic health records (her) systems is between 6% and 13% [3]. While CNSPs were not separated from manufactured products, it is apparent that medication errors occur even in uncomplicated eRx [3]. Moreover, liquid dose form and younger patient age have been identified as significant predictors of prescription errors in pediatric patient populations, with 21.6% and 18.6% of prescriptions containing errors, respectively [4]. A study of 99 compounded drugs dispensed to 71 patients found an average of 3.5 problems per patient, 4% of which involved major real or potential consequences [5]. Another study in adults identified between 30% and 50% of testosterone preparations compounded by ten compounding pharmacies were within ±20% of the prescribed dose [6]. One report from Belgium, a country that in January 2019 made eRx mandatory, indicated that patients with more complex medical conditions were more likely to want paper prescriptions [7]. These outcomes demonstrate the risk for error within every step of compounded drug use and pediatric prescriptions in general, from preparation to administration. 

As the Office of the National Coordinator for Health Information Technology (ONC) recently stated, the 21st Century Cures Act and modern computing may require us to re-examine the assumptions about what the delivery of care should be [8]. “Should be” is an ethical statement, and many of the evolving standards within the health IT landscape will have an impact on provider applications that utilize artificial intelligence algorithms. ONC rulemaking will impact medical management for children in a very positive way with the acceptance of the National Council of Prescription Drug Programs (NCPDP) SCRIPT 2017071 [9]. The NCPDP SCRIPT Standard, first published in 1997, is a resource intended to facilitate the transmission of prescription data between key players and facilities in patient care. Enhancements in the newest version [10] will support mass-based or meter squared dosing, electronic transmission of ingredient compounding information, and reporting of drug allergies, intolerances, and adverse events. Further, the eRx dosing instructions (SigText) limit will be expanded to 1000 characters, and can include the manufacturer name, lot number, and beyond use date. These system enhancements will be available in January 2020 [11]. The NCPDP SCRIPT release will be a paradigm change and take us closer to more complete data transmission for CNSPs. In addition, the Pharmacy Health Information Technology Collaborative (Pharmacy HIT), formed in 2010, has developed goals related to the relationship between clinical pharmacy practice and pediatric patients, with special focus on the transmission of eRx [12]. However, variability in how CNSPs are described at the pharmacy level is a major barrier, and there is an urgent need to codify uniform vocabulary standards at the macro level. United States Pharmacopeia (USP) is vital to this enhanced interoperability through creation of nomenclature standards for the medication management of vulnerable populations, such as children and the elderly. USP’s Expert Panel (EP) on the Exchange of Compounded Drug Preparation Information in Health IT Systems proposes to develop a set of encoding rules that would govern how compounded drug preparations are digitized and exchanged to improve medication management, especially at transitions of care.

## 2. Charge of the Expert Panel

The EP was chartered in October 2017 and convened in February 2018 as a joint panel of the Healthcare Quality and Safety and Compounding Expert Committees. Drs. Richard Parrish and Roy Guharoy co-chair the panel. Included on the panel are pharmacy informatics specialists from children’s hospitals and pharmacy associations and representatives from the National Library of Medicine (NLM), Veterans Administration (VA), United States Food and Drug Administration (FDA), and ONC. The primary intended outcome for encoding CNSPs is the minimization of medication errors caused by the nonstandard transmission of prescription information generated in/on authorized prescribers’ locations/devices for products compounded in licensed pharmacies. Electronic clinical decision support (eCDS) for CNSPs at points of care provision, such as medication reconciliation functions and drug therapy problem identification—that is, drug–diagnosis mismatches, drug–drug interaction assessment, dose–range checking, and drug or constituent allergies, intolerances, and alerts—are virtually nonexistent, although eCDS has been shown to reduce prescribing errors by 36% to 87% [13]. 

The EP was charged with proposing a set of rules that would govern how compounded drug preparations are modeled, encoded, and exchanged in patient electronic health records, pharmacy systems, e-prescribing, and other health IT systems where medication information related to the patient is needed. While not part of the work-product of the EP, these rules could be applied to compounded sterile products (CSPs). An environmental scan was performed to identify CNSPs known to be needed for vulnerable populations, such as infants and children. In addition, a survey designed to gather information from patient care providers will verify the level of information detail necessary for CNSPs at each decision point in the medication use process. That is to say, the metadata needed to understand formulation identification and preparation. Once these tasks are complete, a data model will be derived to encode CNSPs digitally so that an unambiguous name, along with meaningful information connected to that name, for a CNSP can flow from prescription initiation to fulfillment. Coordination with eRx organizations and standards-setting bodies, NCPDP, and the NLM’s Unified Medical Language System (RxNorm) will verify model alignment with existing vocabulary standards. Ultimately, in coordination with other relevant USP Expert Committees on healthcare quality and safety, compounding, and nomenclature, the EP will publish a draft set of encoded CNSP formulation identifiers that can be integrated into health IT systems. 

## 3. Methods

### 3.1. Workplan Overview

#### 3.1.1. Monograph Identification

For monograph sources, the EP compiled a cross-walk of those CNSPs that have been vetted previously or funded through FDA, USP, and American Society of Health-System Pharmacists (ASHP), among others. Table 1 shows the data repositories and sources deemed to be within the scope of the EP’s purview. In addition, the *Essential Medicines for Children* list from the World Health Organization (WHO) and the FDA bulk drug substance list were examined to determine opportunities to expand the number of CNSPs identified. That no CNSPs were listed in WHO’s list provides an opportunity for international cooperation and coordination for scientifically-validated and encoded preparations sponsored through USP and its worldwide partners.

#### 3.1.2. Survey of Professionals

An electronic survey has been prepared to gather input from physicians, compounding pharmacists, pharmacy technicians, and informatics and billing specialists to identify and verify the types of information needed for prescribing and compounding CNSPs. The specificity is likely to relate to the setting, such as the picklists in EHR systems and pharmacy prescription fulfillment systems. In other words, the survey will determine how much detail is needed in eRx generation to make a decision about the use of a CNSP within various locations where care is provided. These locations include hospitals, children’s hospitals, community pharmacies, skilled nursing, and long-term care, and compounding pharmacies. The primary purpose of the survey is to validate and prioritize the interoperability needs for CNSPs across healthcare settings through understanding the clinical workflows and patient experience. Proposed question domains include the patient’s need for a CNSP, complete order entry, transmission, and receipt of the order, quality and safe preparation, Rx transfer process, accurate and safe dose administration, and complete transmission of information between settings. USP has coordinated the selection of the survey vendor, conducted selected pilot studies, finalized and distributed the survey, and will analyze data for a target completion around the end of 2019. By gaining an understanding of the clinical workflow across healthcare settings, the EP will be able to validate and prioritize interoperability needs for their forthcoming standards on encoding and exchange of compounded drug information.

#### 3.1.3. Data Model Development

After receiving input from health professionals regarding the scope of the concepts to be included within the developed codes and standards, the EP will define the discrete data elements that uniquely identify a CNSP name and its monograph. Using data from the first two groups, monograph identification, and survey administration, this group will propose the data elements contained in monographs that need to be digitized in the clinical formulation identifier to assure interoperability in the electronic medium to facilitate accurate CNSP preparation. The data model will be interoperable with other electronic vocabulary standards, such as RxNorm.

### 3.2. Nomenclature Standards for Digitizing CNSPs 

The following section expresses the opinions and insights of the authors and not necessarily those of the EP or USP. The work of USP’s expert committees and panels can provide leadership in an official forum for addressing non-standard product descriptions related to salt form of the active pharmaceutical ingredient (API), standard expression of liquid concentration, inclusion of indication, and identification of the need for multiple strengths for different populations. The USP’s Compounding Compendium is a monograph repository for all CNSPs that could become a part of an encrypted electronic comprehensive medication management (CMM) system for children. This system would be accessible through an authorized portal for verified network participants whose verification is governed through a consortium of national and international organizations and professional associations. To be a USP verified network participant for children’s CMM, a pharmacist might fulfill the following requirements in the form of a profile: 1) evidence of training for CSP and/or CNSP preparation; 2) evidence of training for collection of patient-specific clinical information at the point-of-care (i.e., REDCap or other mechanisms for data collection); 3) evidence of board certification in pediatric pharmacy or a business relationship with a board-certified pharmacist; and 4) evidence of a collaborative practice with a healthcare team or physician.

USP’s compound monographs and pending monographs could function like the structured product labeling (SPL) and target product profiles (TPP) for manufactured drug products [14]. In this way, a systematic determination of whether a substance should be compounded, expansion of the Compounding Compendium, and improvements in children’s access to quality compounded preparations can be achieved.

To derive vocabulary standards for all CSPs and CNSPs, several currently existing USP chapters provide guidance. Compounded preparations need to be named in accordance with Chapter <1121> Nomenclature and their dosage forms named in accordance with Chapter <1151> Pharmaceutical Dosage Forms. The most common forms of oral liquids for pediatric application are solutions and suspensions. As defined in these chapters, solutions are liquid preparations containing one or more drug substances dissolved in a suitable solvent or mixture of mutually miscible solvents. Suspensions are liquid preparations containing drug substance(s) and consist of solid particles dispersed throughout a liquid phase in which the particles are present in excess of the solubility [15]. While the USP verified program relates currently to dietary supplements, the concept of verification could be applied to compounded preparations. Those preparations with a USP monograph could be described as ‘USP verified.’ In the case of CNSPs, the ‘verified’ designation would pertain to the validated process used to create for formulation as specified in the USP monograph.

Digitization could take place via the use of RxNorm for drug-related terms and the systematized nomenclature of medicine-clinical terms (SNOMED-CT) for health-related conditions. SNOMED-CT encodes the meanings of medical conditions that are used in health information and to support the effective clinical recording of data with the aim of improving patient care [16]. Encoding all compounded preparations consists of discreet data elements that form a string of numbers describing extemporaneous products as found in their monographs. The alpha–numeric line item description of the CSPs and CNSPs may contain the standardized API United States Adopted Name (USAN), dosage form, concentration with USP ‘verified’ designation (if applicable), and fixed dispensing quantities. Any new concepts needed to represent compounded drug preparations could have relationships with existing concepts in RxNorm. For example, “120 mL cloNIDine HCL 10 mCg/mL compounded oral suspension, USP” could have a structured relationship back to the RxNorm concept unique identifier (RxCUI) 142432 ‘cloNIDine hydrochloride’ which represents the active pharmaceutical ingredient. Although RxNorm does not currently include nomenclature for compounded drug preparations, one could envision a compounded drug ‘extension’ of RxNorm, which would allow for interoperable exchange of these entities. 

### 3.3. Fee Management

A fee schedule for all CSPs and CNSPs could be negotiated, derived, and published in the same way that other professional and product fees are managed. Groups, such as the NCPDP, Pharmaceutical Care Management Association (PCMA), the Joint Commission of Pharmacy Practitioners (JCCP), and Pharmacy Benefit Management Institute (PBMI), could be involved. If not assigned already, each CNSP would have a healthcare common procedural coding system (HCPCS) code that would set a maximum reimbursement for a given billing unit, a common methodology in which the work-product of other professions is monetized and compensated [17]. While this step may be controversial in pharmacy, it is a common methodology that other professions utilize to monetize and receive compensation for standardized units of their work-product.

## 4. Discussion

The USP Compounding Expert Committee and the USP Healthcare Quality and Safety Expert Committee created a joint expert panel on the exchange of compounded drug preparation information in health IT systems. The goal is to develop a set of rules that govern how compounded drug preparations are encoded and exchanged in patient records, pharmacy systems, e-prescribing, and other Health IT systems where patient medication information is critical. This proposal builds upon and complements existing ideas and architecture provided by NCPDP SCRIPT and Pharmacy HIT to improve the overall landscape of compounded drug preparation information transmission.

Within the scope of compounded drug preparations, practice variance-related errors create unintended care outcomes and potential harm for vulnerable patients, such as children. The disparity in compounded drug preparation between locations threatens to produce inconsistent medications for a patient, which is exacerbated by the existing complexity of compounding for children due to diverse dosage forms, strengths, and routes of administration [18]. Even outside of compounding, pediatric prescription errors are not uncommon and may be the result of calculation errors, using the total daily dose instead of dividing into recommended frequencies, volume-based instead of mass-based doses, and lack of patient weight information needed for dose validation, among others [19,20]. Children, especially CMC, therefore, often face more health and safety risk as a result of complex care needs than as a result of their illness itself [21]. As a science-based, standards-setting organization, USP is well-positioned to address such an important healthcare system need. 

## 5. Conclusions

In summary, the work of the EP is focused in three areas: (1) monograph development expansion and validation; (2) a survey of professionals involved in medication management informatics; and (3) data model development and integration within existing data structures. USP’s coordination and leadership for setting nomenclature-encoding standards have the potential to improve medication management in children, especially at transitions of care. Moreover, an outcomes-focused agenda that seeks to minimize medication errors through the complete transmission of compounded drug preparation information and improved pharmacy specialization in compounding and pediatrics can assure safer medication access and use, both here and abroad. Most importantly, the work addresses a long term unmet need.

## Figures and Tables

**Table 1:** Environmental scan results for compounded nonsterile drug preparations (CNSPs).

Source of Information	Number of CNSPs Identified
Michigan collaborative / other States	104
American Society of Health-Systems Pharmacists	30
United States Pharmacopeia	133
Food and Drug Administration bulk substances list	283
International Journal of Pharmaceutical Compounding	10
Veterans Administration nonsterile compounded oral	98
World Health Organization essential medications/children	0

## Abbreviations

ASHP	American Society of Health-system Pharmacists
API	active pharmaceutical ingredient
CF-ID	clinical formulation identifier
CMC	children with medical complexity
CNSP	compounded nonsterile preparation
CMM	comprehensive medication management
CSHCN	children with special healthcare needs
CSP	compounded sterile preparations
eCDS	electronic clinical decision support
HER	electronic health record
EP	Expert Panel on Exchange of Compounded Drug Preparation Information in Health IT Systems
eRx	electronic prescribing
FDA	United States Food and Drug Administration
HCPCS	Healthcare Common Procedural Coding System
HIT	health information technology
JCCP	Joint Commission of Pharmacy Practitioners
NCPDP	National Council of Prescription Drug Programs
NLM	National Library of Medicine
ONC	Office of the National Coordinator for Health Information Technology
PBMI	Pharmacy Benefit Management Institute
PCMA	Pharmaceutical Care Management Association
Pharmacy HIT	Pharmacy Health Information Technology Collaborative
SNOMED-CT	systematized nomenclature of medicine-clinical terms
RxCUI	RxNorm concept unique identifier
SPL	structured product labeling
TPP	target product profiles
USAN	United States adopted name
USP	United States Pharmacopeia
WHO	World Health Organization
